# Radiomics signature of epicardial adipose tissue for predicting postoperative atrial fibrillation after pulmonary endarterectomy

**DOI:** 10.3389/fcvm.2022.1046931

**Published:** 2023-01-09

**Authors:** Zhan Liu, Yisen Deng, Xuming Wang, Xiaopeng Liu, Xia Zheng, Guang Sun, Yanan Zhen, Min Liu, Zhidong Ye, Jianyan Wen, Peng Liu

**Affiliations:** ^1^Department of Cardiovascular Surgery, Peking University China-Japan Friendship School of Clinical Medicine, Beijing, China; ^2^Department of Cardiovascular Surgery, China-Japan Friendship Hospital, Beijing, China; ^3^Department of Radiology, China-Japan Friendship Hospital, Beijing, China

**Keywords:** radiomics, epicardial adipose tissue, postoperative atrial fibrillation, pulmonary endarterectomy, chronic thromboembolic pulmonary hypertension

## Abstract

**Purpose:**

This study aimed to construct a radiomics signature of epicardial adipose tissue for predicting postoperative atrial fibrillation (POAF) after pulmonary endarterectomy (PEA) in patients with chronic thromboembolic pulmonary hypertension (CTEPH).

**Methods:**

We reviewed the preoperative computed tomography pulmonary angiography images of CTEPH patients who underwent PEA at our institution between December 2016 and May 2022. Patients were divided into training/validation and testing cohorts by stratified random sampling in a ratio of 7:3. Radiomics features were selected by using intra- and inter-class correlation coefficient, redundancy analysis, and Least Absolute Shrinkage and Selection Operator algorithm to construct the radiomics signature. The area under the receiver operating characteristic curve (AUC), calibration curve, and decision curve analysis (DCA) were used to evaluate the discrimination, calibration, and clinical practicability of the radiomics signature. Two hundred-times stratified five-fold cross-validation was applied to assess the reliability and robustness of the radiomics signature.

**Results:**

A total of 93 patients with CTEPH were included in this study, including 23 patients with POAF and 70 patients without POAF. Five of the 1,218 radiomics features were finally selected to construct the radiomics signature. The radiomics signature showed good discrimination with an AUC of 0.804 (95%CI: 0.664–0.943) in the training/validation cohort and 0.728 (95% CI: 0.503–0.953) in the testing cohorts. The average AUC of 200 times stratified five-fold cross-validation was 0.804 (95%CI: 0.801–0.806) and 0.807 (95%CI: 0.798–0.816) in the training and validation cohorts, respectively. The calibration curve showed good agreement between the predicted and actual observations. Based on the DCA, the radiomics signature was found to be clinically significant and useful.

**Conclusion:**

The radiomics signature achieved good discrimination, calibration, and clinical practicability. As a potential imaging biomarker, the radiomics signature of epicardial adipose tissue (EAT) may provide a reference for the risk assessment and individualized treatment of CTEPH patients at high risk of developing POAF after PEA.

## Introduction

Chronic thromboembolic pulmonary hypertension (CTEPH) is characterized by chronic stenosis and occlusion of the pulmonary arteries due to obstructive intraluminal organized thromboembolic material (1, 2). The narrowing and occlusion of proximal pulmonary arteries, in combination with a secondary microvasculopathy, leads to increased pulmonary vascular resistance and progressive right ventricular failure (3). Pulmonary endarterectomy (PEA) is the treatment of choice for operable patients, allowing for major hemodynamic and clinical improvements (3–5).

Postoperative atrial fibrillation (POAF) is a common complication after PEA, with an incidence of about 10–24.8% (6–11). Development of POAF has been previously associated with longer lengths of stay, more postoperative complications (9), reduced functional capacity (10), and worsened health-related quality of life measures (8). Therefore, preoperative identification of patients at high risk of developing POAF is crucial for improving the prognosis and quality of life of CTEPH patients who underwent PEA.

Although the pathogenesis of POAF remains uncertain, accumulating evidence suggests an important role in inflammatory mechanisms and mediators (12, 13). Epicardial adipose tissue (EAT) is a special visceral adipose tissue located between the myocardium and the visceral pericardium (14). It produces numerous pro-inflammatory cytokines which can promote the development of POAF (15, 16). Quantitative analysis of EAT can be performed by cardiac computed tomography (CT). It is reported that the EAT volume and EAT radiodensity were found to be associated with the occurrence, severity, and recurrence of AF (17–20).

Radiomics is a new method in medical imaging analysis that can extract large amounts of image features from radiographic images (21). It can also provide and uncover quantitative disease characteristics that fail to be detected by observational measures (22). Radiomics analysis of EAT has been previously proven to be useful in identifying AF (23, 24), differentiating AF characteristics, and predicting AF recurrence (14). Therefore, radiomics may provide additional information beyond EAT volume and radiodensity.

There are currently no studies on the correlation between EAT and POAF after PEA in terms of CT quantitative analysis and radiomics analysis. Therefore, this study aims to construct a CT-based radiomics signature of EAT for predicting POAF after PEA in patients with CTEPH.

## Materials and methods

### Patient selection

This study was approved by the Ethics Board of China-Japan Friendship Hospital (No. 2019-142-K98) and individual consent for this retrospective analysis was waived. From December 2016 to May 2022, a total of 112 patients with CTEPH underwent PEA in our institution. Of these, 19 patients without available CT pulmonary angiography (CTPA) images were excluded. Finally, 93 patients were enrolled in this study. Considering the small sample size, stratified random sampling was adopted. Patients were grouped based on the clinical outcome (POAF or not) in a ratio of 7:3, with 65 and 28 patients in the training/validation and testing cohorts, respectively.

### Clinical features

Preoperative demographics, electrocardiogram, World Health Organization functional class, 6-min walking distance, hematologic examination, echocardiography, and right heart catheterization examination were collected from the electronic medical record system. All patients underwent continuous electrocardiographic monitoring during the postoperative period and until discharge. POAF was defined as any documented AF episode lasting >30 s recorded either by continuous telemetry throughout the hospitalization stay or on a 12-lead electrocardiogram performed daily and when the patient reported experiencing symptoms (25).

### CTPA examination

All patients underwent CTPA examination within 1 week before surgery. The CTPA examination was performed using a multi-detector CT system (Philips iCT/256; Lightspeed VCT/64, GE Healthcare, Milwaukee, WI, USA; Toshiba Aquilion ONE TSX-301C/320; Siemens Sensation/16, SOMATOM Definition Dual Source CT). The CT scan parameters were as follows: tube voltage, 100–120 kV; tube current, 100–300 mAs; rotation time, 0.8 s; matrix, 512 × 512; section thickness, 0.625–1 mm; and reconstruction increment, 1–1.25 mm.

### EAT segmentation

Epicardial adipose tissue segmentation for radiomics analysis was performed with 3D slicer software (version 4.13.0). Volumes of interest (VOIs) were manually delineated independently by two experienced radiologists along the margins of the visceral pericardium on cardiac axial slices, from pulmonary artery bifurcation to cardiac apex. The two radiologists were blinded to the patients’ clinical features. A segmentation algorithm based on a densitometric threshold (density range between −190 HU and −30 HU) was used to identify EAT. After the delineation was completed, the EAT volume and radiodensity were automatically calculated by the 3D slicer software. One month later, reader 1 repeated the delineation of VOIs in all patients. The average of the EAT volume and radiodensity of three separate measurements were recorded.

### Feature extraction and selection

PyRadiomics (version 3.0.1) and Python (version 3.7) were used for radiomics feature extraction from the VOIs with image normalization and resampling. Wavelet transform and Laplacian of Gaussian filters were applied to the image. Finally, a total of 1,218 quantitative radiomics features were extracted from each VOI of the original images and their corresponding transform-filtered images.

Feature selection was performed in the training/validation cohort. The extracted radiomics features in the training/validation cohort were normalized to eliminate differences introduced by value scales between features. The features in the testing cohort were normalized based on the mean value and standard deviation derived from the training/validation cohort. Intra-class and inter-class correlation coefficients (ICC) were used to assess the reproducibility of the radiomics features. Features with an ICC of greater than 0.75 were considered to have agreeable reproducibility and were chosen for further analysis. The Spearman or Pearson correlation coefficients for each pair of features were calculated, and redundant features with a correlation coefficient of more than 0.9 were removed. Then the Least Absolute Shrinkage and Selection Operator (LASSO) algorithm was applied to identify significant radiomics features with non-zero coefficients based on the selected features. The penalty parameter (λ) was optimized by 10-fold cross-validation *via* minimum criteria.

### Radiomics signature construction and evaluation

**FIGURE 1:**
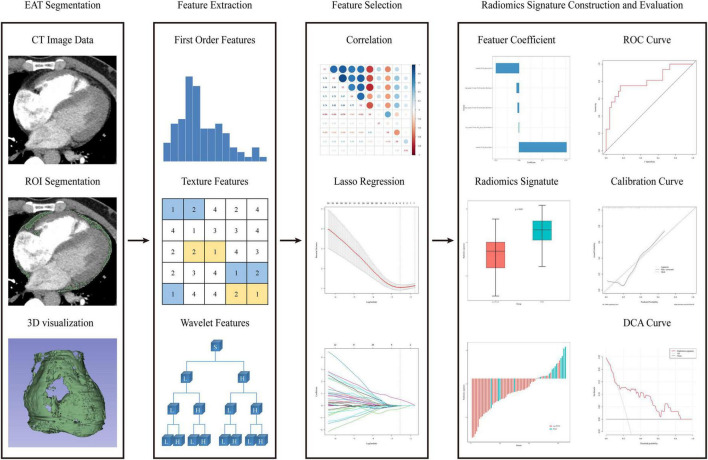
Flowchart of the radiomics analysis process.

The selected features from VOIs were analyzed by linear regression model and weighted by their respective coefficients to build a radiomics signature. The discrimination was evaluated by the area under the receiver operating characteristic curves (AUC). In order to avoid over-optimized estimation, 200 times stratified five-fold cross-validation was applied. In the stratified five-fold cross-validation, the patients in the training/validation cohort were grouped based on the clinical outcome (POAF or not) in a ratio of 4:1 for 5 times. During each-time validation, the validation cohort was used to evaluate the discrimination of the radiomics signature. This process was replicated 200 times and the average AUC was used to assess the reliability and stability of the constructed radiomics signature. The Hosmer–Lemeshow test and calibration curves were performed to assess the calibration. The accuracy of the radiomics signature in the calibration curves was assessed by bootstrap validation with 1,000 resamplings. In addition, decision curve analysis (DCA) was adopted to evaluate the clinical practicability by quantifying the net benefit at different threshold probabilities. The whole process of radiomics analysis can be completed in a few minutes in R software. R code used for radiomics analysis can be obtained from the following website: https://github.com/bubble0405/EAT-radiomcs. The flowchart of the radiomics analysis process is shown in Figure 1.

### Statistics analysis

R software (version 3.5.1) and SPSS (version 26.0, IBM Corp., Armonk, NY, USA) were used for statistical analyses. The “irr” package was used to calculate the ICC. The “sampling” package was adopted for stratified random sampling. The LASSO algorithm was performed by the “glmnet” package. The “rms” package was used to construct the logistic regression model. The “pROC,” “ggplot2,” and “rmda” packages were adopted to plot the ROC curve, calibration curve, and DCA curve. The Hosmer–Lemeshow test was performed by the “ResourceSelection” package.

The Kolmogorov–Smirnov test was used to test the normality of continuous variables. Continuous variables consistent with a normal distribution were presented as mean ± standard deviation, otherwise, the median and interquartile range were used. Categorical variables were presented as a number (percentage). The independent-sample *t*-test or Mann–Whitney *U* test was employed to evaluate the statistical differences in continuous variables, while the Chi-Square test or Fisher’s exact test was used to compare the categorical variables. Two-sided *P* < 0.05 indicated a significant difference.

## Results

### Clinical characteristics

A total of 93 patients with CTEPH were retrospectively included in this study, including 23 patients with POAF (24.7%) and 70 patients without POAF. No significant differences were found in clinical features between the training/validation and testing cohorts (Table 1). Furthermore, in both training/validation and testing cohorts, we did not find any significant difference in clinical features, EAT volume, or EAT radiodensity (Table 2; Supplementary Table 1). However, in the whole cohort, age (*P* = 0.017) and EAT volume (*P* = 0.043) were significantly different between patients with and without POAF (Supplementary Table 2). The multivariate analysis revealed that patient age (Odds ratio [OR] = 1.049, 95% confidence interval [CI]: 1.005–1.049, *P* = 0.029) was an independent predictor for POAF after PEA with an AUC of 0.667 (95% CI: 0.541–0.793, *P* = 0.017) (Supplementary Figure 1).

**TABLE 1:** Clinical characteristics in training/validation and testing cohorts.

Characteristic	Training/validation cohort (*n* = 65)	Testing cohort (*n* = 28)	*P*-value
Gender (*n*,%)			0.263
Male	41 (63.1)	21 (75.0)	
Female	24 (36.9)	7 (25.0)	
Age (years)	53.0 (42.0,60.0)	57.5 (41.0, 63.0)	0.497
Body mass index (kg/m^2^)	24.3 ± 3.9	24.1 ± 3.0	0.819
Smoking history (*n*,%)	15 (23.1)	11 (39.3)	0.110
Resting heart rate (bmp)	82.6 ± 16.2	80.2 ± 16.9	0.530
6MWD (m)	390.0 (310.0, 470.0)	391.0 (290.0, 477.3)	0.728
WHO functional class (*n*,%)			0.843
I	2 (3.1)	1 (3.6)	
II	33 (50.8)	12 (42.9)	
III	22 (33.8)	12 (42.9)	
IV	8 (12.3)	3 (10.7)	
**Comorbidity (*n*,%)**			
Hypertension	12 (18.5)	6 (21.4)	0.740
Diabetes mellitus	2 (3.1)	0 (0)	1.000
Coronary artery disease	7 (10.8)	5 (17.9)	0.550
Dyslipidemia	11 (16.9)	4 (14.3)	0.992
**Hematologic examination**			
WBC (×10^9^/L)	5.4 (4.7, 6.7)	5.8 (4.6, 7.1)	0.429
C-reactive protein (mg/L)	3.0 (2.5, 4.2)	2.5 (2.5, 5.9)	0.943
Potassium (mmol/L)	4.1 ± 0.4	4.1 ± 0.5	0.637
Total cholesterol (mmol/L)	3.7 ± 1.3	3.7 ± 0.8	0.971
NT-proBNP (pg/ml)	502.5 (113.0, 1,266.8)	754.0 (67.0, 1,590.5)	0.672
**Echocardiogram**			
LA diameter (mm)	34.4 ± 4.7	36.2 ± 6.3	0.135
LV diameter (mm)	42.2 ± 5.4	41.5 ± 6.0	0.598
LVEF (%)	69.0 (65.0, 73.0)	69.0 (64.3, 73.0)	0.895
RA diameter (mm)	48.5 (42.3, 58.5)	49.5 (44.0, 63.0)	0.473
RV diameter (mm)	47.0 (42.3, 52.8)	45.5 (43.3, 54.8)	0.763
**Right cardiac catheterization**			
Cardiac index (L/min/m^2^)	1.8 (1.5, 2.2)	1.7 (1.6, 2.2)	0.954
RA pressure (mmHg)	8.0 (5.0, 12.0)	8.0 (5.0, 10.0)	0.986
RV pressure (mmHg)	25.8 ± 7.2	27.7 ± 7.5	0.268
Mean PA pressure (mmHg)	41.6 ± 11.3	43.6 ± 11.4	0.423
PVR (dyn⋅s⋅cm^–5^)	821.7 (542.3, 1,071.9)	823.7 (602.7, 1,198.3)	0.702
EAT volume (ml)	94.3 (70.2, 138.8)	118.3 (89.1, 136.3)	0.156
EAT radiodensity (HU)	−95.3 ± 4.7	−95.6 ± 6.0	0.745
POAF (*n*,%)	16 (24.6)	7 (25.0)	0.969

EAT, epicardial adipose tissue; LA, left atrium; LV, left ventricle; LVEF, left ventricular ejection fraction; PA, pulmonary artery; POAF, postoperative atrial fibrillation; PVR, pulmonary vascular resistance; RA, right atrium; RV, right ventricle; WBC, white blood cell; WHO, World Health Organization; 6MWD, 6-min walking distance.

**TABLE 2:** Univariate analysis of risk factors for POAF in the training/validation cohort.

Characteristic	POAF (*n* = 16)	Non-POAF (*n* = 49)	*P*-value
Gender (*n*,%)			0.065
Male	7 (43.8)	34 (69.4)	
Female	9 (56.3)	15 (30.6)	
Age (years)	55.5 (50.3, 62.3)	50.0 (39.5, 60.0)	0.073
Body mass index (kg/m^2^)	24.8 ± 4.1	24.1 ± 3.8	0.522
Smoking history (*n*,%)	1 (6.3)	14 (28.6)	0.134
Resting heart rate (bmp)	87.1 ± 19.2	81.1 ± 15.0	0.196
6MWD (m)	388.0 (333.0, 450.0)	400.0 (300.0, 473.8)	0.790
WHO functional class (*n*,%)			0.638
I	1 (6.3)	1 (2.0)	
II	8 (50.0)	25 (51.0)	
III	6 (37.5)	16 (32.7)	
IV	1 (6.3)	7 (14.3)	
**Comorbidity (*n*,%)**			
Hypertension	3 (18.8)	9 (18.4)	1.000
Diabetes mellitus	1 (6.3)	1 (2.0)	0.990
Coronary artery disease	1 (6.3)	6 (12.2)	0.836
Dyslipidemia	3 (18.8)	8 (16.3)	1.000
**Hematologic examination**			
WBC (×10^9^/L)	6.1 (4.5, 7.6)	5.3 (4.7, 6.6)	0.180
C-reactive protein (mg/L)	3.1 (2.5, 13.7)	3.0 (2.5, 4.1)	0.476
Potassium (mmol/L)	4.1 ± 0.3	4.0 ± 0.4	0.528
Total cholesterol (mmol/L)	3.6 ± 1.3	3.8 ± 1.4	0.779
NT-proBNP (pg/ml)	479.0 (61.0, 978.0)	551.0 (115.0, 1,437.0)	0.500
**Echocardiogram**			
LA diameter (mm)	35.3 ± 4.7	34.1 ± 4.7	0.391
LV diameter (mm)	43.3 ± 6.2	41.8 ± 5.1	0.345
LVEF (%)	67.5 (65.3, 73.5)	69.0 (63.0, 73.0)	0.920
RA diameter (mm)	47.0 (40.0, 55.0)	49.5 (43.0, 61.3)	0.356
RV diameter (mm)	44.5 (39.5, 49.8)	47.5 (43.0, 53.8)	0.142
**Right cardiac catheterization**			
Cardiac index (L/min/m^2^)	1.8 (1.4, 2.3)	1.8 (1.5, 2.2)	1.000
RA pressure (mmHg)	7.5 (6.0, 11.3)	8.0 (5.0, 12.0)	0.669
RV pressure (mmHg)	24.6 ± 7.0	26.1 ± 7.3	0.477
Mean PA pressure (mmHg)	38.1 ± 9.8	42.7 ± 11.6	0.151
PVR (dyn⋅s⋅cm^–5^)	619.8 (413.0, 1,308.5)	881.2 (590.4, 1,071.9)	0.285
EAT volume (ml)	127.2 (78.3, 170.6)	89.0 (67.8, 117.2)	0.059
EAT radiodensity (HU)	−96.4 ± 5.4	−94.9 ± 4.4	0.261

EAT, epicardial adipose tissue; LA, left atrium; LV, left ventricle; LVEF, left ventricular ejection fraction; PA, pulmonary artery; POAF, postoperative atrial fibrillation; PVR, pulmonary vascular resistance; RA, right atrium; RV, right ventricle; WBC, white blood cell; WHO, World Health Organization; 6MWD, 6-min walking distance.

### Feature selection and radiomics signature construction

**FIGURE 2:**
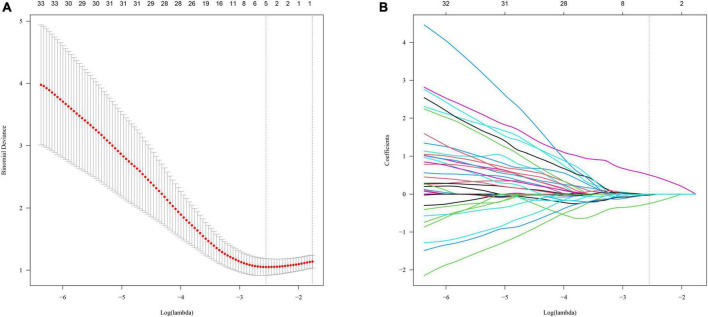
The Least Absolute Shrinkage and Selection Operator (LASSO) algorithm was used to select radiomics features. The penalty parameter (λ) was optimized by 10-fold cross-validation *via* minimum criteria **(A)**. LASSO coefficient profiles **(B)**.

A total of 1,218 radiomics features were extracted from each VOI by using PyRadiomics. Using an ICC of 0.75 as a cut-off value, 1,136 radiomics features with good reproducibility were selected for further analysis (Supplementary Figure 2). Subsequently, redundant features with a Spearman or Pearson correlation coefficient of more than 0.9 were eliminated. After applying the LASSO algorithm (Figure 2), five of 213 radiomics features were finally selected and used to construct the radiomics signature (Supplementary Figure 3). The detailed formula used to create the radiomics signature is shown in Supplementary Formula 1. The radiomics signature distributions of each patient in the two cohorts are shown in Supplementary Figure 4. In patients with POAF, the value of radiomics signature was significantly higher than non-POAF patients in the training/validation cohort (−1.38 ± 0.55 vs. −0.70 ± 0.55, *P* < 0.001) but not in the testing cohort (−1.31 ± 0.62 vs. −0.84 ± 0.41, *P* = 0.073) (Supplementary Figure 5).

### Radiomics signature evaluation

**FIGURE 3:**
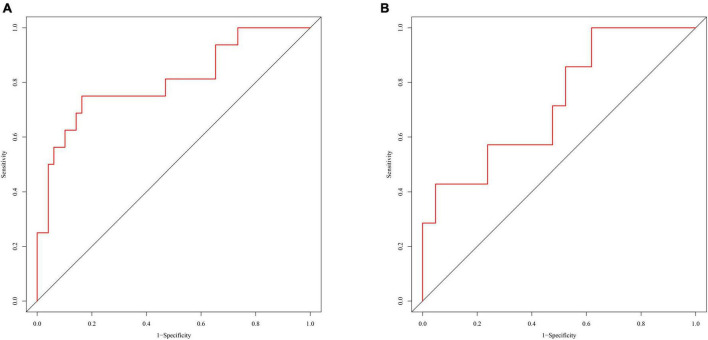
Receiver operating characteristic curves of the radiomics signature in the training/validation cohort **(A)** and testing cohort **(B)**.

**FIGURE 4:**
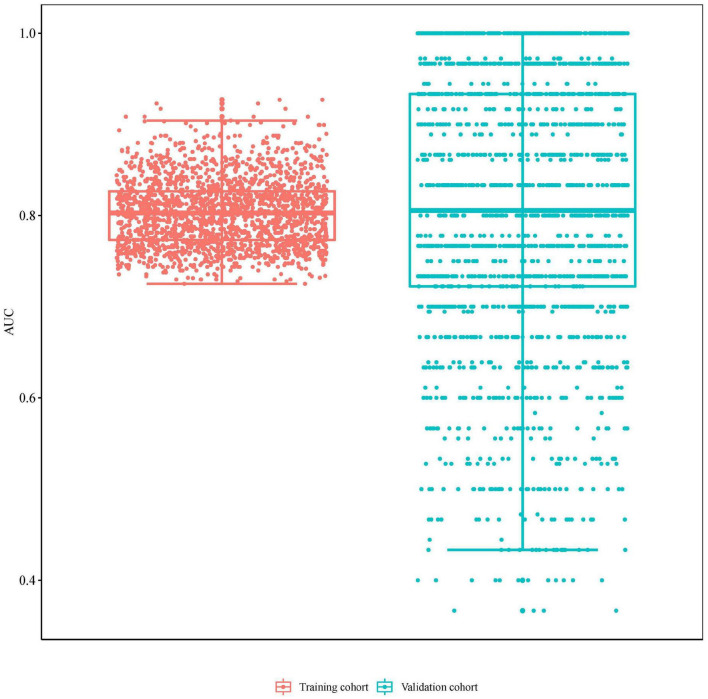
The area under the receiver operating characteristic curve (AUC) value of 200 times stratified five-fold cross-validation in the training and validation cohorts.

**FIGURE 5:**
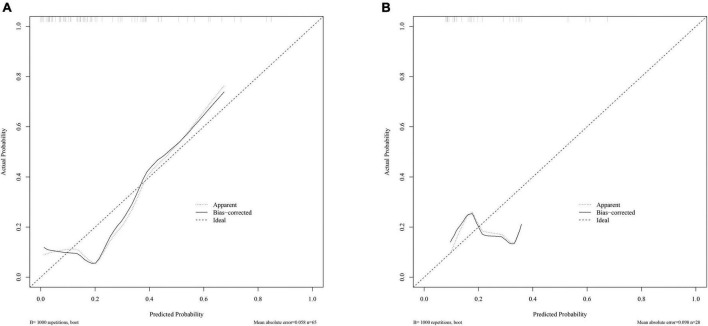
Calibration curves of the radiomics signature in the training/validation cohort **(A)** and testing cohort **(B)**.

**FIGURE 6:**
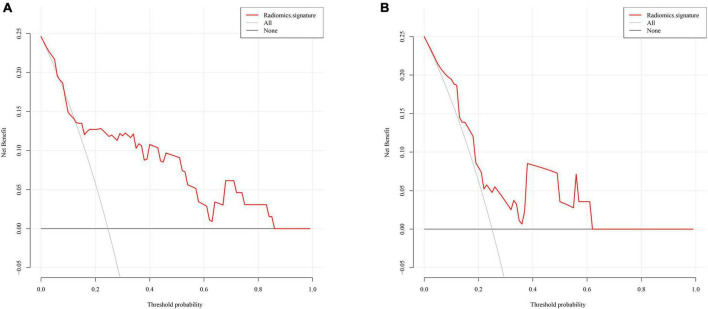
Decision curve analysis for the radiomics signature in the training/validation cohort **(A)** and testing cohort **(B)**.

The radiomics signature showed good discrimination with an AUC of 0.804 (95% CI:0.664–0.943) in the training/validation cohort and 0.728 (95% CI:0.503–0.953) in the testing cohorts (Figure 3). The average AUC of 200 times stratified five-fold cross-validation was 0.804 (95%CI: 0.801–0.806) and 0.807 (95%CI: 0.798–0.816) in the training and validation cohorts, respectively (Figure 4). The calibration curve revealed good agreement between the predicted and actual observations in the training/validation and testing cohorts (Figure 5). In addition, the Hosmer–Lemeshow test demonstrated no statistical significance in the training/validation (*P* = 0.173) and testing cohorts (*P* = 0.632), which indicated no departure from a perfect fit. As shown in Figure 6, the radiomics signature could provide a higher net benefit than the “treat-all” and “treat-none” schemes, with a threshold probability of 13–85% for the training/validation cohort and 1–61% for the testing cohort. Based on the DCA, the radiomics signature was found to be clinically significant and useful.

## Discussion

In this study, based on the radiomics features of EAT, we first constructed and validated a radiomics signature for the individual preoperative prediction of POAF in CTEPH patients who underwent PEA. The radiomics signature showed good discrimination, calibration, and clinical practicability in the training/validation and testing cohorts.

POAF represents a common event complicating the postoperative course of 20–40% of patients undergoing cardiac surgery (26). Recent publications have highlighted the association between POAF and increased mortality and morbidity (27, 28). PEA, a very specialized cardiac surgery entailing median sternotomy, cardiopulmonary bypass, and deep hypothermic circulatory arrest, is currently the only method that could effectively cure of CTEPH (3–5). The incidence of POAF after PEA has been reported to be approximately 10–24.8% (6–11), less than that of common cardiac surgery. However, POAF after PEA was associated with longer lengths of stay, more postoperative complications (9), reduced functional capacity (10), and worsened health-related quality of life measures (8). Therefore, preoperative identification of patients at high risk of developing POAF is critical for improving the prognosis and quality of life of CTEPH patients who underwent PEA. Unfortunately, only a few studies have investigated preoperative clinical predictors for POAF after PEA. Age, male sex, prior atrial arrhythmias, baseline right atrial pressure, and resting heart rate were reported as independent predictors for POAF after PEA (9, 11). Advanced age has consistently been recognized as an independent predictor of POAF after cardiac surgery and non-cardiac surgery (29–31). In our study, age was the only independent predictor for POAF after PEA in the whole cohort with an AUC value of 0.667. Patients with POAF were older than those without POAF in both the training/validation and testing cohorts, but no significant differences were found. This may be related to the random assignment of samples and the small sample size. In addition, we did not find any differences between patients with and without POAF in terms of functional class, hematologic examination, echocardiography, and right heart catheterization examination. This suggests that clinical features provide limited usefulness in predicting POAF after PEA.

Epicardial adipose tissue is a special visceral adipose tissue located between the myocardium and the visceral pericardium (14). This privileged location positions EAT to exert important paracrine and vasocrine effects on neighboring cardiomyocytes (32). In pathological contexts, EAT switches from an anti-inflammatory phenotype to a pro-inflammatory phenotype, mediated in part by the release of fatty acids and pro-inflammatory adipokines, including interleukin-1b, interleukin-6, activin-A, and tumor necrosis factor-alpha (33). These pro-inflammatory cytokines have been reported to be associated with POAF after cardiac surgery (15, 16). To a certain extent, the EAT volume and radiodensity reflect its pathological status. It is reported that EAT volume and radiodensity were found to be associated with the occurrence, severity, and recurrence of AF (17–20, 24). Across our whole cohort, the EAT volume was significantly different between patients with and without POAF, but not so much so that it was an independent predictor according to multivariate analysis. Moreover, EAT volume and radiodensity were higher in patients with POAF than in those without POAF, but there were no statistical differences. Therefore, the ability of EAT volume and radiodensity in predicting POAF after PEA needs to be confirmed by further studies.

Radiomics quantitatively assesses tissue heterogeneity, which is an objective measure but not visually recognizable, by reflecting the distribution of gray level values and spatial arrangement of the pixels (34). Radiomics analysis of EAT has been previously shown to be useful in identifying AF (23, 24), differentiating AF characteristics, and predicting AF recurrence (14). These studies revealed that radiomics analysis of EAT may have the potential to provide an accurate prediction for POAF after PEA. In the present study, eight of 1,218 CT-based radiomics features of EAT were selected to construct a radiomics signature. The results revealed that the radiomics signature yielded good discrimination, calibration, and clinical practicability in training/validation and testing cohorts. Furthermore, in order to avoid over-optimized estimation, 200 times stratified five-fold cross-validation was applied. The average AUC of 200 times stratified five-fold cross-validation was 0.804 (95%CI: 0.795–0.813) in the training cohort and 0.807 (95%CI: 0.798–0.816) in the validation cohort, which indicated that the constructed radiomics signature of EAT was a reliable and robust predictor for POAF. To the best of our knowledge, the present study is the first study to construct a radiomics signature of EAT in the prediction of POAF after PEA. A recent study, however, did find that the radiomics profile of EAT could predict POAF in aortic valve replacement patients (24). This suggests that the radiomics signature of EAT can potentially be used to identify patients at high risk of developing POAF. It should also be noted that all five selected radiomics features were transform-filtered features, which was similar to previous studies (35, 36). These transform-filtered features may have the potential to suppress noise and highlight details in the original images, thus extracting areas with increasingly coarse texture patterns in a more flexible way (36). The radiomics features of EAT reflect its histological heterogeneity (14) and may provide additional information beyond the traditional CT quantitative assessment of EAT.

There were some limitations to this study. First, retrospective studies have inherent weaknesses and potential. Further prospective studies are needed to validate our radiomics signature. Second, this was a single-center study with a small sample size. Therefore, more patients from multiple centers could be used to validate the robustness and repeatability of the radiomics signature constructed in this study and further explore the relationship between EAT quantitative features and POAF after PEA. Third, the EAT was segmented manually, which could lead to artificial differences. An accurate, automatic segmentation method should be considered in future studies.

## Conclusion

To sum up, we first constructed a CT-based radiomics signature of EAT in the prediction of POAF after PEA. The radiomics signature achieved good discrimination, calibration, and clinical practicability. As a potential imaging biomarker, the radiomics signature of EAT may provide a reference for the risk assessment and individualized treatment of CTEPH patients at high risk of developing POAF after PEA.

## Data availability statement

The raw data supporting the conclusions of this article will be made available by the authors, without undue reservation.

## Ethics statement

The studies involving human participants were reviewed and approved by the Ethics Board of China-Japan Friendship Hospital. Written informed consent for participation was not required for this study in accordance with the national legislation and the institutional requirements.

## Author contributions

ZL, YD, and XW: the first draft of the manuscript. XL, XZ, YZ, GS, ML, and ZY: material preparation and data collection and analysis. JW and PL: conceptualization and revision of the manuscript. All authors commented on previous versions of the manuscript, read, and approved the final manuscript.
