# Supplementary material for: Radiomics signature of epicardial adipose tissue for predicting postoperative atrial fibrillation after pulmonary endarterectomy
Source: Front Cardiovasc Med. 2023 Jan 9;9:1046931. doi: 10.3389/fcvm.2022.1046931 (PMC9869069; doi:10.3389/fcvm.2022.1046931)
Supplement: Supplementary file 1 [file Data_Sheet_1.docx]

**Supplementary Table 1. Univariate analysis of risk factors for POAF in the testing cohort.**

| Characteristic | POAF  (n=7) | Non-POAF  (n=21) | P value |
| --- | --- | --- | --- |
| Gender (n, %) |  |  | 0.801 |
| Male | 6(85.7) | 15(71.4) |  |
| Female | 1(14.3) | 628.6) |  |
| Age (years) | 63.0(56.0,64.0) | 49.0(37.0,62.5) | 0.123 |
| Body mass index (kg/m^2^) | 25.8±3.8 | 23.5±2.6 | 0.089 |
| Smoking history (n, %) | 3(42.9) | 8(38.1) | 1.000 |
| Resting heart rate (bmp) | 86.0±23.0 | 78.3±14.5 | 0.304 |
| 6MWD (m) | 399.5(338.8,416.5) | 377.5(259.0,503.55) | 0.951 |
| WHO functional class (n, %) |  |  | 0.877 |
| I | 0(0) | 1(4.8) |  |
| Ⅱ | 4(57.1) | 8(38.1) |  |
| Ⅲ | 3(42.9) | 9(42.9) |  |
| Ⅳ | 0(0) | 3(14.3) |  |
| **Comorbidity** (n, %) |  |  |  |
| Hypertension | 3(42.9) | 3(14.3) | 0.144 |
| Diabetes mellitus | 0(0) | 0(0) | - |
| Coronary artery disease | 0(0) | 5(23.8) | 0.290 |
| Dyslipidemia | 2(28.6) | 2(9.5) | 0.253 |
| **Hematologic examination** |  |  |  |
| WBC (*10^9^/L) | 5.8(5.1,7.8) | 5.8(4.2,7.0) | 0.710 |
| C-reactive protein (mg/L) | 2.5(2.5,2.5) | 4.0(2.5,5.97) | 0.281 |
| Potassium (mmol/L) | 4.1±0.4 | 4.1±0.5 | 0.797 |
| Total Cholesterol (mmol/L) | 3.9±0.6 | 3.7±0.9 | 0.752 |
| NT-proBNP (pg/ml) | 754.0(385.0,1413.8) | 755.0(58.5,1689.0) | 0.947 |
| **Echocardiogram** |  |  |  |
| LA diameter (mm) | 35.7±4.8 | 36.4±6.8 | 0.813 |
| LV diameter (mm) | 42.0±6.5 | 41.4±6.0 | 0.818 |
| LVEF (%) | 72.0(68.0,74.0) | 68.0(64.0,73.0) | 0.298 |
| RA diameter (mm) | 47.0(42.0,50.0) | 54.0(44.5,65.5) | 0.117 |
| RV diameter (mm) | 44.0(42.0,52.0) | 47.0(44.0,56.5) | 0.276 |
| **Right cardiac catheterization** |  |  |  |
| Cardiac index (L/min/m^2^) | 1.8(1.6,2.2) | 1.7(1.5,2.2) | 0.626 |
| RA pressure (mmHg) | 8.0(6.0,10.0) | 8.0(4.3,10.8) | 0.738 |
| RV pressure (mmHg) | 27.9±7.1 | 27.6±7.9 | 0.935 |
| Mean PA pressure (mmHg) | 45.4±9.6 | 43.1±12.1 | 0.642 |
| PVR (dyn·s·cm^-5^) | 850.0(766.3,1156.8) | 821.4(561.3,1279.4) | 0.626 |
| EAT volume (ml) | 105.7(103.3,133.0) | 120.0(85.5,138.5) | 0.730 |
| EAT density (HU) | -95.1±7.1 | -95.8±5.8 | 0.786 |

EAT, epicardial adipose tissue; LA, left atrium; LV, left ventricle; LVEF, left ventricular ejection fraction; PA, pulmonary artery; POAF, postoperative atrial fibrillation; PVR, pulmonary vascular resistance; RA, right atrium; RV, right ventricle; WBC, white blood cell; WHO, World Health Organization; 6MWD, 6-minute walking distance.

**Supplementary Table 2. Univariate analysis of risk factors for POAF in the whole cohort.**

| Characteristic | POAF  (n=23) | Non-POAF  (n=70) | P value |
| --- | --- | --- | --- |
| Gender (n, %) |  |  | 0.234 |
| Male | 13(56.5) | 49(70.0) |  |
| Female | 10(43.5) | 21(30.0) |  |
| Age (years) | 58.0(51.0,63.0) | 49.5(39.8,60.0) | 0.017 |
| Body mass index (kg/m^2^) | 25.1±4.0 | 23.9±3.5 | 0.174 |
| Smoking history (n, %) | 4(17.4) | 22(31.4) | 0.193 |
| Resting heart rate (bmp/min) | 86.8±19.9 | 80.2±14.8 | 0.095 |
| 6MWD (m) | 390.0(335.0,447.5) | 390.0(296.3,478.8) | 0.794 |
| WHO functional class (n, %) |  |  | 0.603 |
| Ⅰ | 1(4.3) | 2(2.9) |  |
| Ⅱ | 12(11.1) | 33(47.1) |  |
| Ⅲ | 9(39.1) | 25(35.7) |  |
| Ⅳ | 1(4.3) | 10(14.3) |  |
| **Comorbidity** (n, %) |  |  |  |
| Hypertension | 6(26.1) | 12(17.1) | 0.346 |
| Diabetes mellitus | 1(4.3) | 1(1.4) | 0.402 |
| Coronary artery disease | 1(4.3) | 11(15.7) | 0.293 |
| Dyslipidemia | 5(21.7) | 10(11.3) | 0.606 |
| **Hematologic examination** |  |  |  |
| WBC (*10^9^/L) | 5.8(5.0,7.6) | 5.4(4.7,6.7) | 0.158 |
| C-reactive protein (mg/L) | 2.5(2.5,4.4) | 3.0(2.5,4.4) | 0.841 |
| Potassium (mmol/L) | 4.1±0.3 | 4.1±0.4 | 0.515 |
| Total Cholesterol (mmol/L) | 3.7±1.1 | 3.7±1.2 | 0.864 |
| NT-proBNP (pg/ml) | 526.0(83.0,1018.5) | 551.0(112.0,1514.0) | 0.612 |
| **Echocardiogram** |  |  |  |
| LA diameter (mm) | 35.4±4.6 | 34.8±5.5 | 0.632 |
| LV diameter (mm) | 42.9±6.2 | 41.7±5.3 | 0.364 |
| LVEF (%) | 69.0(66.0,74.0) | 69.0(64.0,73.0) | 0.557 |
| RA diameter (mm) | 50.0(45.0,62.0) | 48.0(41.5,58.5) | 0.267 |
| RV diameter (mm) | 44.7(42.0,50.0) | 47.0(43.0,54.5) | 0.336 |
| **Right cardiac catheterization** |  |  |  |
| Cardiac index (L/min/m^2^) | 1.8(1.6,2.2) | 1.7(1.5,2.1) | 0.786 |
| RA pressure (mmHg) | 8.0(6.0,10.0) | 8.0(5.0,12.0) | 0.806 |
| RV pressure (mmHg) | 25.6±7.0 | 26.6±7.4 | 0.615 |
| Mean PA pressure (mmHg) | 40.3±10.1 | 42.8±11.6 | 0.355 |
| PVR (dyn·s·cm^-5^) | 801.8(444.3,1168.8) | 826.0(575.7,1081.0) | 0.483 |
| EAT volume (ml) | 118.0(97.3,151.3) | 93.6(76.1,132.0) | 0.043 |
| EAT density (HU) | -95.2±4.8 | -96.0±5.8 | 0.493 |

EAT, epicardial adipose tissue; LA, left atrium; LV, left ventricle; LVEF, left ventricular ejection fraction; PA, pulmonary artery; POAF, postoperative atrial fibrillation; PVR, pulmonary vascular resistance; RA, right atrium; RV, right ventricle; WBC, white blood cell; WHO, World Health Organization; 6MWD, 6-minute walking distance.

**Supplementary Formular 1**

The radiomics signature was calculated as follows:

Radiomics signature =

- 1.210625157

- 0.025492217 * log.sigma.5.0.mm.3D_firstorder_Skewness

+ 0.507143986 * wavelet.LLH_glcm_Idmn

- 0.247073454 * wavelet.HLH_glcm_Imc2

- 0.015273790 * log.sigma.2.0.mm.3D_firstorder_Skewness

- 0.002072263 * log.sigma.2.0.mm.3D_glcm_ClusterShade

**Supplementary Figure 1.** Receiver operating characteristic curve of age in prediction POAF. The AUC was 0.667 (95% CI: 0.541 - 0.793, *P* = 0.017).

AUC, areas under ROC curve; POAF, postoperative atrial fibrillation.

**Supplementary Figure 2**. Scatter plot of inter-class correlation coefficient (A) and intra-class correlation coefficient (B). X-axis shows the individual radiomic features (n=1218), while Y-axis shows the ICC value for each radiomic feature. The red horizontal line indicates ICC=0.75. Features with good reproducibility (ICC > 0.75) were selected for further analysis.

ICC, inter-class or intra-class correlation coefficient.

**Supplementary Figure 3.** Selected features and corresponding coefficients.

**Supplementary Figure 4.** Bar plots for he radiomics signature of each patient in the training/validation cohort (A) and testing cohort (B). The orange bars indicate the radiomics signatures for patients with POAF, while the blue bars present the radiomics signatures for those without POAF. The horizontal axis indicates the cut-off value of radiomics signature.

POAF, postoperative atrial fibrillation.

**Supplementary Figure 5.** The boxplots of the radiomics signature in the training/validation cohort (A) and testing cohort (B).
