# Supplementary figures and images for: Radiomics signature of epicardial adipose tissue for predicting postoperative atrial fibrillation after pulmonary endarterectomy
Source: Front Cardiovasc Med. 2023 Jan 9;9:1046931. doi: 10.3389/fcvm.2022.1046931 (PMC9869069; doi:10.3389/fcvm.2022.1046931)

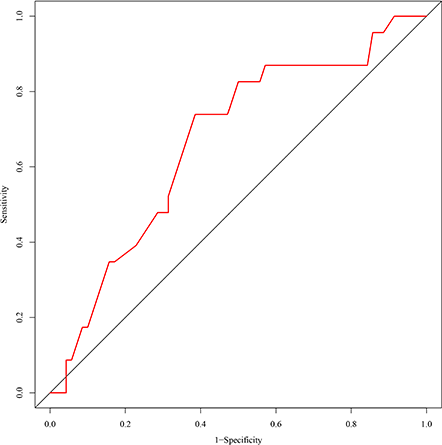

Supplement: Supplementary file 2 [file Image_1.TIFF]

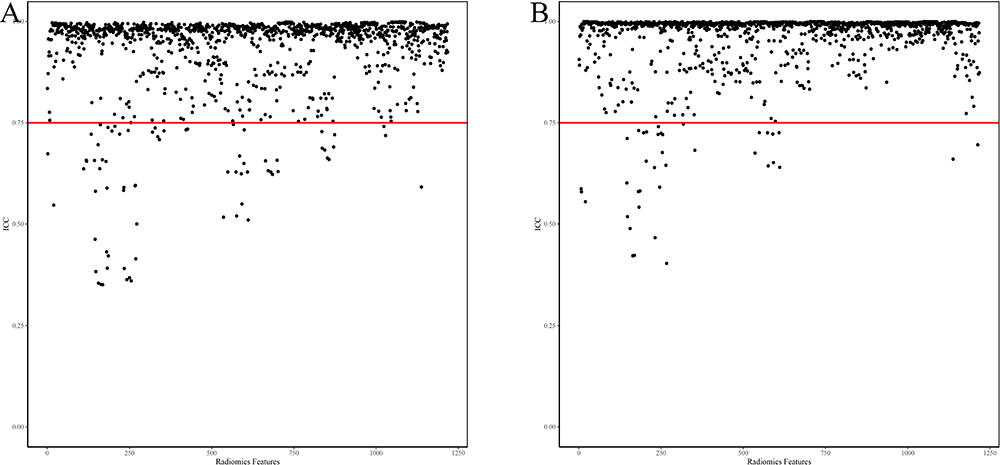

Supplement: Supplementary file 3 [file Image_2.TIFF]

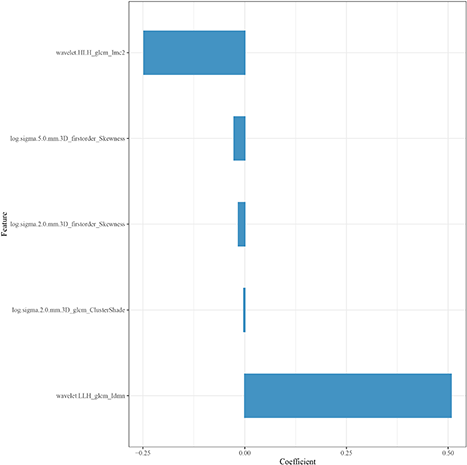

Supplement: Supplementary file 4 [file Image_3.TIFF]

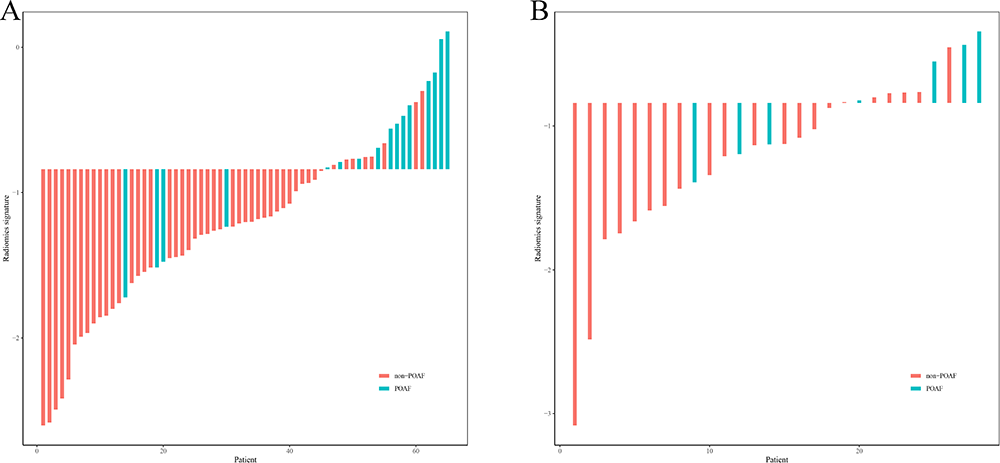

Supplement: Supplementary file 5 [file Image_4.TIFF]

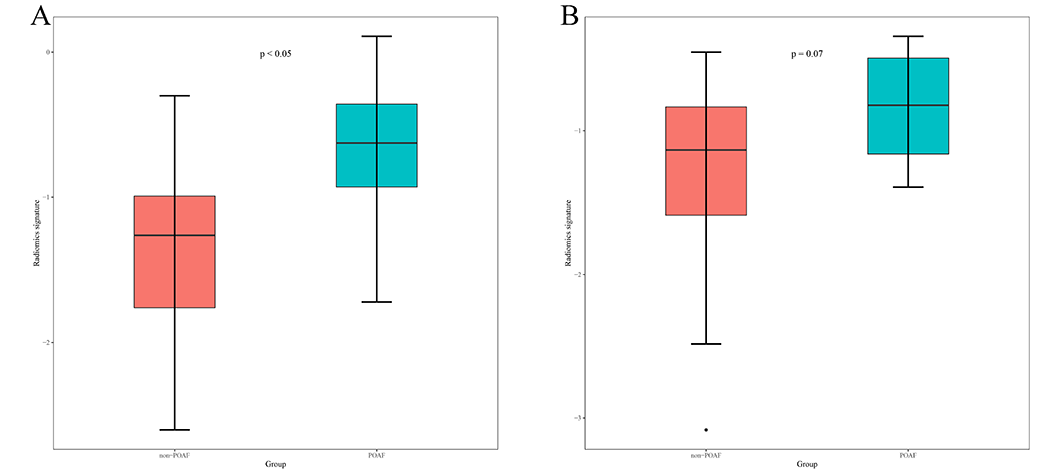

Supplement: Supplementary file 6 [file Image_5.TIFF]
